# Automated VOI Analysis in FDDNP PET Using Structural Warping: Validation through Classification of Alzheimer's Disease Patients

**DOI:** 10.1155/2012/512069

**Published:** 2012-03-01

**Authors:** Moses Q. Wilks, Hillary Protas, Mirwais Wardak, Vladimir Kepe, Gary W. Small, Jorge R. Barrio, Sung-Cheng Huang

**Affiliations:** ^1^Department of Biomathematics, David Geffen School of Medicine at UCLA, Box 951766, Los Angeles, CA 90095, USA; ^2^Department of Molecular and Medical Pharmacology, David Geffen School of Medicine at UCLA, Box 951735, Los Angeles, CA 90095, USA; ^3^Department of Psychiatry and Biobehavioral Sciences, David Geffen School of Medicine at UCLA, Los Angeles, CA 90095, USA

## Abstract

We evaluate an automated approach to the cortical surface mapping (CSM) method of VOI analysis in PET. Although CSM has been previously shown to be successful, the process can be long and tedious. Here, we present an approach that removes these difficulties through the use of 3D image warping to a common space. We test this automated method using studies of FDDNP PET in Alzheimer's disease and mild cognitive impairment. For each subject, VOIs were created, through CSM, to extract regional PET data. After warping to the common space, a single set of CSM-generated VOIs was used to extract PET data from all subjects. The data extracted using a single set of VOIs outperformed the manual approach in classifying AD patients from MCIs and controls. This suggests that this automated method can remove variance in measurements of PET data and can facilitate accurate, high-throughput image analysis.

## 1. Introduction

In the field of quantitative imaging, the creation of accurate volumes of interest (VOIs) is often of central importance. This process, however, can be time-consuming and is known to have variance introduced on inter- and intra-investigator levels. Various approaches have been employed to reduce the time and labor involved and the noise variance in the definition of VOIs [1, 2]. Most of these approaches try to map the images of individuals to a reference image in a common space. They vary on the mapping methods and on the selection of the common space and the reference image. A discussion of various methods and approaches is provided later in this section [2–4]. The choice of approach depends on the type of images (i.e., MRI or PET) and the desired VOIs one is considering. An approach that has been previously shown to be effective in accomplishing the spatial normalization of PET images is the use of hemispheric cortical surface mapping [2, 5]. However, the process involved is complex and labor intensive. An improved procedure suited for automated and streamlined operation is thus warranted. In this paper, we introduce a modified method that can reduce the variance in VOI analysis by warping structural and functional images to a common space in which valid VOIs already exist. This method is also easily adaptable to an automated approach to VOI analysis.

There have been previous attempts at similar methods, by creating maximum likelihood estimates (MLEs) of VOIs in a stereotaxic space [1]. In these methods, VOIs are manually drawn on several brains, which are normalized to a stereotaxic brain space such as the International Consortium for Brain Mapping (ICBM53) average space [6]. After a subject's brain image has been normalized to this space, MLEs can be used to create an individualized VOI based on the population of manually drawn VOIs. One drawback to using these methods is that when normalizing a subject's brain to the common stereotaxic space, it is difficult to balance how closely to match the target and template images. If the images are not matched closely enough, the MLEs for creating individualized VOIs lose their validity. However, if images are matched too closely to an average image of multiple brains, the structure of the subject's brain image can be lost when the normalization algorithm mistakes noise in the common space population as actual structural information. To reduce as much of the investigator-based variance as possible, it would seem ideal to use a library of previously created VOIs in a high-resolution single brain common space. There have been efforts to create such libraries [7] however, the problem then becomes finding a way to closely normalize a subject's image to this single common space while maintaining its structural integrity.

Currently, there are many nonlinear methods available for spatial normalization that use a wide array of image matching methods. Some of these methods use a set of smooth basis functions [3], while another large algorithm family contains the nonparametric methods such as Diffeomorphic Demons [8], ART [9], or SyN [4]. Additionally, there are methods such as the DARTEL algorithm in SPM8 [10] that use a fluid deformation model that simultaneously matches gray matter to gray matter, and white matter to white matter. Recently, fourteen such algorithms were tested against one another in MRI brain registration by Klein et al. [11]. The evaluation was done using 80 manually labeled MRI brain images and eight separate measures of performance, in which the SyN algorithm was ranked the overall best. The rankings of each algorithm were relatively consistent across image sets, labeling protocols, and image matching metrics. Klein et al. believe this is strong evidence that these rankings can be generalized to other sets of subjects and labels. The SyN algorithm has the benefit of creating diffeomorphic deformations, so that there is no shearing or tearing of the image being deformed. Additionally, the SyN algorithm is not limited to imaging modalities that can be accurately segmented into gray and white matter, so it can be used in studies using non-T1-weighted MRIs. Therefore, we opted to use the SyN algorithm in our investigation of automated VOI analysis.

We apply this method to a set of MRI images that have corresponding PET images of the tracer, 2-(1-6-[(2-[^18^F]fluoroethyl)(methyl)amino]-2-naphthylethylidene)malononitrile (FDDNP), which can bind to the cerebral *β*-amyloid plaques and neurofibrillary tangles (NFTs) characteristic of Alzheimer's disease (AD). The FDDNP PET images were obtained from a study of AD and mild cognitive impairment (MCI) patients. Patients classified as MCI do typically have a larger decline in cognitive function than normal aging, especially in memory, but do not show clinical signs of dementia [12]. Moreover, MCI patients do have a greatly increased chance of developing dementias such as AD over the non-MCI population [13]. The primary neuropathological characteristic of AD is accumulation of *β*-amyloid plaques and NFTs across numerous cortical regions. This accumulation of these misfolded protein aggregates follows a pattern of deposition described by Braak and Braak [14], in which different cortical regions are affected at varying severity throughout the progression of the disease. Braak and Braak proposed three stages (A–C) for the deposition of *β*-amyloid, and six stages (I–VI) for the pattern of deposition of NFTs.

Although AD is clinically recognizable at late stages due to characteristic dementia and decline in cognitive abilities, current clinical diagnosis standards do not yet produce definitive differentiation between AD and non-Alzheimer's dementias (e.g., frontotemporal dementia) [15, 16]. However, current evidence shows that the accumulation of the neuropathological markers begins long before the onset of clinically recognizable symptoms [17]. In *postmortem *studies, cognitively normal controls (clinical dementia rating (CDR) 0) have been shown to display density and distribution of *β*-amyloid and NFTs similar to mildly demented (CDR 0.5) patients [18]. In another *postmortem *study, Peterson et al. reported clinically healthy controls whose NFT distribution spanned stages 0–V in the Braak and Braak progression, with 25% of these controls presenting NFTs consistent with at least stage III [19]. In the same study, Peterson et al. showed that once clinical symptoms are present, the Braak pathology stages do show correlation with the severity of symptoms. Of the MCI patients studied, 80% presented with Braak NFT stages II–IV and 91% of the AD patients presented with stages IV–VI [19].

In both living patients, as well as in postmortem determinations, FDDNP signal has been shown to reside in areas with high *β*-amyloid plaque and NFT loads [2]. Along with the known spatial and temporal pattern of deposition, this suggests that molecular imaging using FDDNP PET may be a powerful tool in early detection and diagnosis of AD. For these same reasons, this compound is a good candidate for the evaluation of automated regional VOI analysis. In this work, we validated the method of structural warping by examining the spatial overlap between the common space image and the warped MRIs in specific structures and regions. Furthermore, we validate the automated approach by comparing the efficacy of VOI data extracted from warped and unwarped functional images to create discriminant models classifying subjects as normal controls, MCI, or AD.

## 2. Methods

### 2.1. Subjects

The study group was comprised of 7 AD (76 ± 10 years, 4 : 3 female/male), 6 MCI (73 ± 13 years, 4 : 2 female/male), and 10 control (71 ± 10 years, 7 : 3 female/male) subjects. Subjects were classified into groups using the diagnostic criteria for AD and amnestic MCI [2]. Subjects who had some memory symptoms but did not meet the diagnostic criteria for either disease group were classified as controls. No subject included in this study had a history of stroke, head injury, or non-Alzheimer's disease which would affect cognitive function. All subjects (*n* = 23) were given mini-mental state examinations (MMSE) to assess cognitive abilities. AD patients had an average MMSE score of 23 ± 2, MCIs had an average score of 27 ± 1, and controls had an average score of 29 ± 1. This subject population has been previously described by Protas et al. [2].

### 2.2. Imaging

A T1-weighted gradient echo (MP-RAGE) image was taken for each subject with a 3T Siemens Allegra MRI scanner (sagittal plane; repetition time (TR) 2300 ms; echo time (TE) 2.93 ms; 160 slices; slice thickness 1 mm; in-plane voxel size 1.3 × 1.3 mm; field of view 256 × 256; flip angle 8°) [2]. FDDNP was produced as described elsewhere [20], and each subject was given a bolus injection of FDDNP (320–550 MBq). A dynamic PET scan was taken for up to 125 min (six 30s frames; four 180s frames; five 600s frames; three 1200s frames). Imaging was performed using an ECAT EXACT HR+ scanner (Siemens Corp.). The images were reconstructed using filtered backprojection with attenuation correction. After the initial reconstruction, a movement correction algorithm was applied [21, 22]. This algorithm corrects for motion artifacts introduced during the 125-minute scan. Each emission frame is aligned with the transmission frame and then reconstructed using the proper attenuation coefficients.

### 2.3. Creation of VOIs

All MRIs were normalized using the cortical surface mapping method. By this method, a 9-parameter linear registration is applied to bring all subjects into rough alignment with a common space, in this case the International Consortium for Brain Mapping (ICBM53) common space [6]. A 3D model of each subject's cortical surface (in the ICBM space) was extracted from their respective MRIs through the use of a method previously described by Thompson et al. [5]. On this model, 36 sulci major sulci and fissures were manually identified on each cortical surface, after which the surface was flattened to a 2D cortical surface map. The previously identified sulci and fissures were then redrawn on the flat map. These sulci were matched to an average 2D sulcal map through a nonlinear deformation. Inverting the flattening procedure and applying this deformation brought the 3D cortical surfaces of all subjects into close alignment. On the average cortical surface, nine ROIs were drawn bilaterally over the following regions: upper parietal lobe, posterior frontal lobe, prefrontal lobe, occipital-parietal lobes, posterior temporal lobe, upper temporal lobe, lower temporal lobe, medial temporal lobe, and the posterior-cingulate gyrus. These ROIs were projected into 3D VOIs by including each voxel within 9 mm of the originally drawn voxels on the cortical surface. The inverse of the cortical surface mapping method for each subject was applied to these VOIs, such that each subject's MRI aligned to the ICBM space had an individualized set of these VOIs [2]. In addition, MRIs in the ICBM space were segmented into white and gray matter using the automated segmentation algorithm in SPM8 [23].

### 2.4. Creation of FDDNP-DVR Images

Logan analysis was performed on the movement-corrected PET images to create FDDNP distribution volume ratio (DVR) images [21]. Following the procedure described previously by Kepe et al. [24] and Small et al. [25], the cerebellar cortex was used as a reference region to approximate an input function. The first six minutes of the FDDNP scan—representing a perfusion image—were summed (frames 1–7), and a ROI was drawn over cerebellar cortex in that summed image. This ROI was then used to extract data from each individual frame, creating a time activity curve (TAC). Using this TAC as the input function for Logan analysis [26], the DVR value for each voxel was set to the slope of the respective Logan plot over the frames from 15 minutes to the end of the scan [2]. A rigid, linear transformation was calculated using SPM2 [23], to align the early summed FDDNP frames (0–6 minutes), to the MP-RAGE image. This transformation was then applied to DVR images, to bring it into alignment with the MRI [2].

### 2.5. Image Warping

Central to the method of automated VOI analysis is the ability to bring the imaging data of all subjects in close alignment with a common space. To accomplish this goal, we used the symmetric image normalization method (SyN) described by Avants et al. [4], as implemented in the software package ANTs [27]. The SyN algorithm creates a diffeomorphic mapping (i.e., one that is both invertible and differentiable) along a geodesic path between a target image *I* and a template image *J*. SyN takes advantage of the fact that such diffeomorphisms can be decomposed into two parts, *φ*_1_ and *φ*_2_, such that the mapping *φ*(*I*) = *φ*_2_^−1^(*φ*_1_(*I*)) = *J*. This allows for the symmetry of the algorithm so that regardless of whether the image is the “target” or “template,” the same deformation is computed. The subfunctions are defined such that the magnitudes of the deformations they define are equal, and that *I* and *J* contributed equally to the deformation. The SyN algorithm can create such a mapping with several different optimization metrics, but for our purposes, images were matched using localized cross-correlation (CC). This metric is a measure of local image mean and variance. It is computed over 3D windows, on the order of 5^3^ voxels. Briefly, the algorithm sets up a global maximization of CC, which is translated into a series of Euler-Langrage equations which are then solved subject to several constraints. As stated above, the two sub-deformations must contribute equally and be both invertible and differentiable. The solutions are solved iteratively. These iterations are carried out at multiple levels of resolution. At each level, computation continues until convergence, or a maximum number of iterations is reached.

### 2.6. Analysis

**Figure 1:**
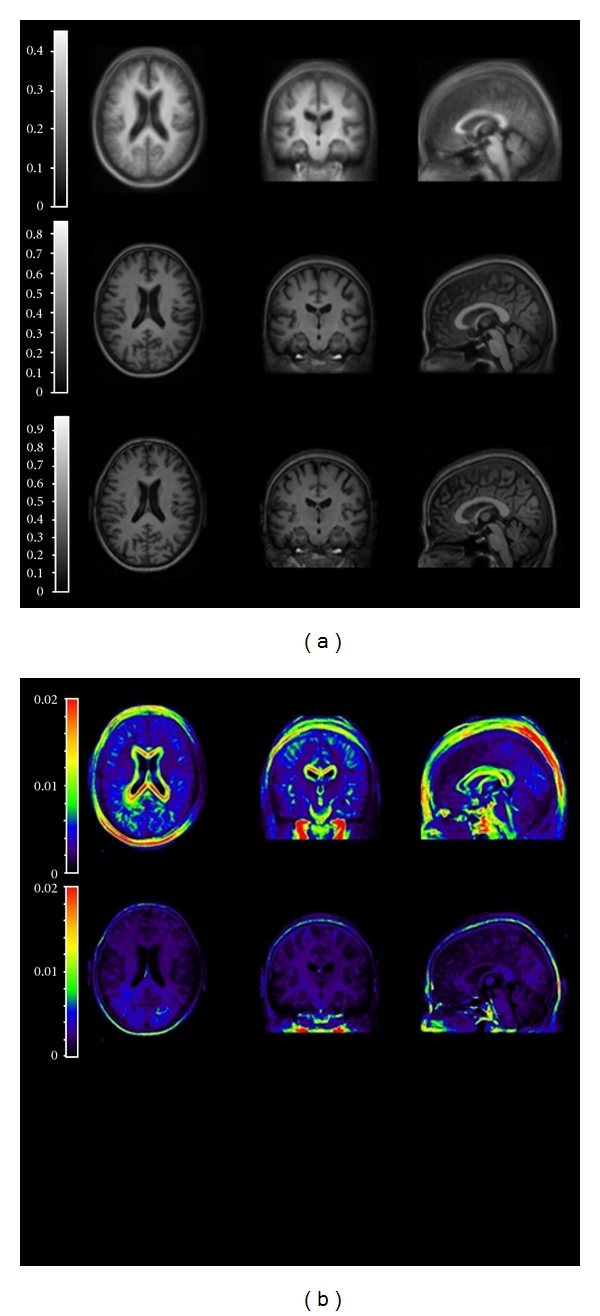
(a) (Left) Warping results: average of 22 subjects MRIs after linear registration only (top); average of 22 subjects after warping to common space (middle); MRI of common space subject (bottom). (b) (Right) Absolute voxel-to-voxel variance of unwarped (top) and warped (bottom) MRIs. Warping reduces average in-brain variance by 54% from linear registration alone.

One control subject was designated as the common space (Figure 1(a) bottom), to which all other subjects would be warped. For each remaining subject's MRI scan, a three-dimensional diffeomorphic warp was calculated to normalize it to the common space. This warp was computed to maximize cross-correlation in windows of size 4^3^ voxels, between the common space and subject images. The software ANTs employs a multiscale resolution approach to image warping. We chose to use four levels of resolution with scaling factors [1, 2, 4, 8], with the maximum iteration number set for each level as (100, 100, 50, 20) This warp was applied to all previously drawn VOIs, segmentation images, and coregistered FDDNP-DVR images for that subject. For each subject, the average FDDNP-DVR values in each of the nine VOIs were measured. This measurement was first performed using a subject's VOI to extract data from the respective unwarped PET image (unwarped data). Next, average DVR values were extracted from warped PET images using VOIs created for the common space MRI (warped data). The warping algorithm was evaluated using the Dice overlap statistic, *κ*, calculated between the regions of the common space image and the regions of subjects warped into the common space

(1) $$\kappa =2\times \frac{\#\left(A\cap B\right)}{\left[\#\left(A\right)+\#\left(B\right)\right]}.$$

This measure has a range of [0,1] and captures the overlap between two regions, *A* and *B*. The # (X) operator returns the number of voxels contained in region X. Although there is no way to determine statistical significance of this measure in this context, some investigators consider good results to be *κ* > 0.6 for smaller structures and *κ* > 0.8 for larger structures [4].

Using the statistical software SPSS (SPSS 15.0 for Windows), discriminant analysis was performed to classify subjects into three groups (control, MCI, or AD). In addition to classifying subjects solely on MMSE scores, an exhaustive search of two classes of models was performed. In the first class, models were built using unwarped FDDNP-DVR data extracted from all possible combinations of VOIs; the second was built similarly, except using warped FDDNP-DVR data extracted using the common space VOIs. In both classes, models were built with and without the use of MMSE as a predictor variable. Models in all categories were ranked by classification ability and leave-one-out cross-validation. It is possible that the exhaustive search for best discriminant models led to some survivorship bias. To correct for this possible artifact, we performed a permutation test on the best models found. In this test, the null hypothesis is that there is no underlying structure to the data, and permuting the data labels (control, AD, or MCI) would have no effect on the ability to classify subjects into their respective groups. The group labels were randomized, keeping the distribution of groups the same (10 control, 7 AD, 6 MCI). For each randomization, linear discriminant analysis was performed on the permuted data, and the correct classification percentage was calculated. This was repeated 100,000 times for each of the best models initially discovered. The significance level is then determined as the percentage of randomized models that are performed as well or better than the correctly labeled data [28].

## 3. Results

**Figure 2:**
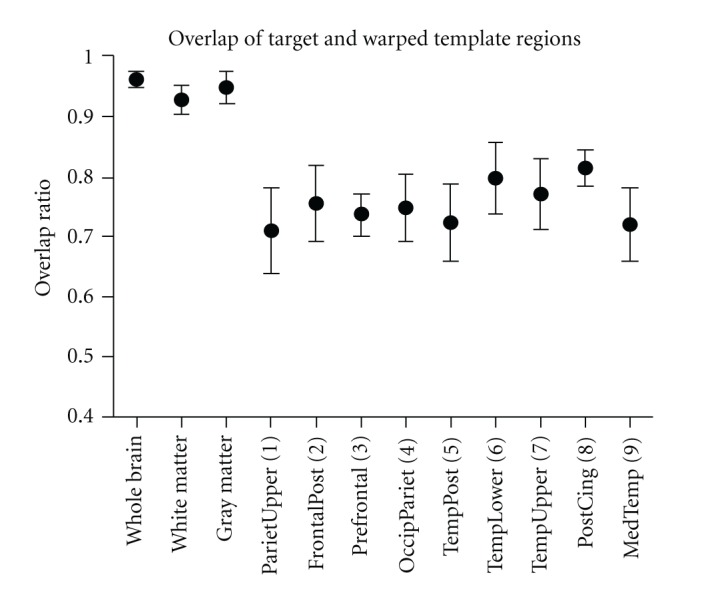
Overlap statistic by region. Data shown is average overlap, ±SD, between common space regions and warped regions of remaining 22 subjects.

Figure 1(a) shows results of the SyN warping algorithm. The average of 22 subject MRIs registered to common space with nine-parameter linear registration alone (top) and with the SyN algorithm (middle) is shown alongside the common space (23rd subject's) MRI (bottom). Images warped to the common space showed a 54% reduction in average absolute voxel-to-voxel variance within the brain (excluding skull and scalp) as compared to variance measured after a nine-parameter linear registration alone (Figure 1(b)). The average (±SD) overlap ratio, *κ*, measured between structures in the common space and those of the 22 remaining subjects warped into the common space are shown in Figure 2. In addition to those for the nine VOIs, overlap between subjects and the common space was also measured for white matter, gray matter, and whole brain structures. In this case, the whole brain is defined as all voxels contained within the cortical surface, as calculated by Protas et al. [2].

**Figure 3:**
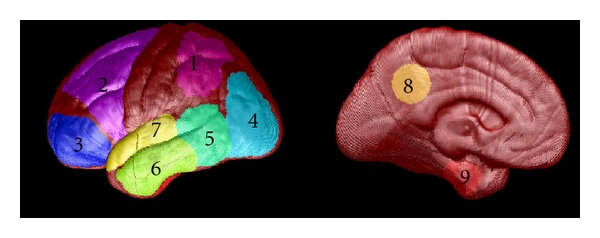
Generalized image of the VOIs used to extract FDDNP data. (Reprinted from Protas et al. 2010 [2]).

**Figure 4:**
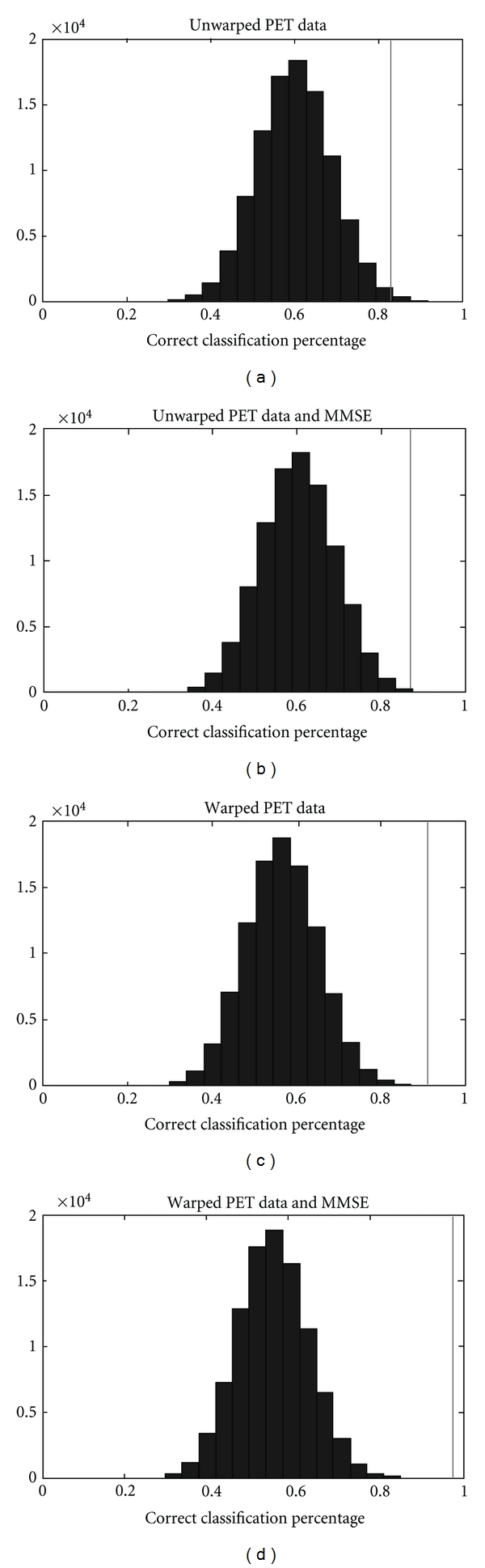
Classification percentages of permutation test. Data shown are for the models using (a) unwarped data only, (b) unwarped data and MMSE, (c) warped data only, and (d) warped data and MMSE. The vertical line represents the score of the true data.

Table 1 shows the best results of the discriminant analysis carried out in SPSS. The models shown are those that had the highest leave-one-out cross-validation scores of all the possible discriminant models in their respective classes. The table shows the classification performance using the original sample (*n* = 23) and with leave-one-out cross-validation. The numbered regions indicated in Table 1 and Figure 2 correspond to those represented in Figure 3. All models shown use FDDNP-DVR data from the occipital-parietal region, the posterior temporal lobe, and the posterior cingulate gyrus. FDDNP-DVR data from the medial temporal lobe is also used in all but one of the models shown. Histograms of the classification percentage of models yielded by the permutation test are shown in Figure 4. Each histogram in Figure 4 also shows the classification percentage of the true, nonpermuted data.

## 4. Discussion

As can be seen in Figure 1, the SyN algorithm is a powerful tool in normalizing a set of structural images. The set of brains, including some with severe cortical degeneration, were mapped almost exactly to a common space, with the borders between sulci and gyri very clearly maintained. The strength of this method is reinforced by the overlap data shown in Figure 2. Although there is no way of describing statistical significance of the overlap statistic in this particular situation, we do see average *κ* > 0.7 for each of the VOIs, which are relatively small structures and only project 9 mm deep into cortex. It is also conceivable that some of the variation is due to the original creation of the VOIs. Although the VOIs were created from a single set drawn on the average cortical surface map, the registration of MRIs to that space was still dependent on manual steps where inter- or intrainvestigator variation could have been introduced. Therefore, it is possible that some of the missed overlap in these VOIs is due to the imperfect nature of the original cortical surface mapping. In addition, the fact that we see *κ* > 0.9 for white and gray matter is especially noteworthy, as we are warping the MRIs of some AD patients with severe cortical degeneration. It is nontrivial that a mapping that preserved sulcus/gyrus boundaries would also maintain cortical gray/white matter boundaries. It is likely that the preservation of these boundaries is due to the use of CC metric. Since the metric is based on matching local variances, as long as tissue types are distinguished by the imaging modalities used, the boundaries between these tissues are likely to be very strictly maintained. Thus, not only does SyN successfully match images visually, but micro- and macrostructures are preserved and matched as well. These results show that the SyN algorithm is an excellent tool for this specific task of image matching. It requires minimal user input, which allows for high-throughput automation and minimizes variance introduced by the investigator. Also, in investigations where one needs to measure the exact spatial distribution of a tracer, an algorithm such as SyN performs quite well because images are matched while maintaining structural boundaries without shearing or tearing.

Table 1 shows that data extracted from FDDNP-DVR images using a single set of VOIs in a common space outperforms data extracted using individual VOIs for each subject in classifying subjects as control, MCI, or AD. Table 1 also shows that the use of FDDNP-DVR can improve classification over using MMSE alone. In addition, the models using warped data use fewer predictor variables than those using unwarped data. Table 1 and Figure 4 show that discrimination between subject classes is likely not a result of survivorship bias, as the *P* value for all models is very low, and for the model using warped data and MMSE, not one of the 100,000 permutations of data labels resulted in a model performing as well as the true data. It is possible that this is a result of decreased noise in measurement of PET data due to standardization of VOI analysis, as with fewer predictor variables we are fitting less noise. There is a reduction in the performance of models when looking at cross-validation scores compared to simple classification, although this could likely be a result of the sample size of this study. Here, we had a sample of 23 subjects, so for cross-validation the models are built with 22 subjects classified into three subpopulations. In this situation, a small amount of misclassification can lead to a large percentage drop in accuracy. It should also be noted that all the discriminant models shown in Table 1 use FDDNP-DVR information from similar regions and from those closely associated with the classical pathological progression of AD, as described by Braak and Braak [14]. 

All models use data from the occipital-parietal region, the posterior temporal lobe, and the posterior cingulate gyrus. The occipital-parietal VOI includes regions of the basal isocortex where initial deposits of amyloid plaques are found, with increasing deposition in stages B and C. This region also shows large amounts of NFTs in late stages of the disease. The posterior temporal lobe and posterior cingulate gyrus both see initial amyloid deposition in stage B with increasing deposition in stage C. Like the occipital-parietal region, these areas also show large amounts of NFT deposition in late stages of the disease. Many of the models also use data from the medial temporal lobe, which is the major site of accumulation for NFTs [14]. Although this is a region canonically associated with the pathological markers of AD, it is possible that it was replaced by MMSE as a predictor variable because FDDNP binding and MMSE share predictive strength for disease state. Giannakopoulos et al. [29] have described that NFT density in the entorhinal cortex is a strong predictor of MMSE in elderly subjects. Thus, it is possible that for this VOI, MMSE and DVR values supply redundant information. As discussed previously, deposition of plaques and NFTs in the medial temporal lobe occurs quite early in the progression of the disease, even before the appearance of clinical symptoms. Therefore, it is also possible that this region might be a weaker predictor of discrimination between noncontrol states (AD and MCI), as elevated FDDNP-DVR values in the medial temporal lobe are present in control subjects and plateau for these non-control subjects. It should also be noted that no special weighting was put on any regions in the search for the best discriminant models.

Central to the utility of this method for further study is the ability to create meaningful and accurate VOIs in a common space for use across data sets. In this paper, we have only used VOIs created via cortical surface mapping and projection into cortex. This procedure has the benefit of creating VOIs that adhere strictly to well-defined cortical regions and can also be tailored to different studies by projecting different depths into cortex depending on the desired application. This method also gains strength when combined with a tool such as the SyN algorithm which, as shown above, strongly matches tissue morphology between subjects. An obvious drawback to this method is its inability to define VOIs over noncortical regions, such as the amygdala or thalamus. However, for well-defined neurological structures such as those, manual creation of such VOIs can be carried out with relative ease and added to a library of VOIs covering a high-resolution common space MRI.

Given the success of this warping method with respect to the classification of subjects with neurological disease, we believe that this can be expanded to a wide variety of applications. First and foremost, it can be used to facilitate accurate high-throughput PET image analysis, by greatly reducing the time needed to extract regional information, while removing several sources of experimental variance. In these applications, almost the entire process of data collection can be streamlined into an automated procedure for clinical applications. Given predefined reference region VOIs, methods such as Patlak or Logan analysis can by automated and normalized, reducing the manual work load required for such methods, and removing variance caused by *ad hoc* definitions of reference regions. In a similar fashion, such a method could be applied to facilitate the use of image-derived input functions for kinetic PET imaging. Once experimentally appropriate analysis has been performed on the raw PET data, the process of regional data extraction can be automated entirely, as shown above. Moreover, this method can be used to facilitate multicenter trials, by using identical image analysis and data extraction across many subjects, investigators, and locations.

## 5. Conclusions

We have shown that the use of the SyN algorithm is a powerful tool for automated image normalization that maintains good alignment of biologically important structures of the warped images. We have also shown that by normalizing data to a common space and using a set of VOIs predrawn in that space, one can actually improve the predictive quality of data extracted from functional images. In addition to being able to better classify subjects into their diagnostic groups, we can do so using fewer predictor variables. This is perhaps due to an elimination of intra-investigator and inter-subject variability by using a single set of VOIs. These results seem to suggest that with a larger sample of subjects with AD and non-Alzheimer's dementias, a protocol could be developed to greatly increase the ability to clinically diagnose patients into their proper groups based on differences in the binding patterns. These results also suggest that automation of VOI analysis through spatial normalization to a single common space brain image can be used to streamline accurate, high-throughput PET image analysis for use in clinical settings. This method is expected to be applicable to longitudinal studies of cognitive impairment as well as to other PET tracers (e.g., other probes for AD imaging), but further study is warranted.

## Figures and Tables

**Table 1:** Best discriminant models.

Model	Classification %	Cross-validation %	Regions used	Permutation significance
MMSE only	77.3	77.3	N/A	N/A
Unwarped PET data only	82.6	73.9	2,4,5,8,9	*P* = 3.42∗10^−3^
Warped PET data only	87	73.9	4,5,8,9	*P* = 1.3∗10^−4^
Unwarped PET data and MMSE	91.3	87	4,5,8,9	*P* = 8.6∗10^−4^
Warped PET data and MMSE	100	95.7	4,5,8	*P* < 10^−5 ^^†^

^†^(None of the 100,000 permutations resulted in a model that performed as well as the true data).
